# The Cerebral Brain-Derived Neurotrophic Factor Pathway, Either Neuronal or Endothelial, Is Impaired in Rats with Adjuvant-Induced Arthritis. Connection with Endothelial Dysfunction

**DOI:** 10.3389/fphys.2017.01125

**Published:** 2018-01-09

**Authors:** Martin Pedard, Aurore Quirié, Philippe Garnier, Anne Tessier, Céline Demougeot, Christine Marie

**Affiliations:** ^1^INSERM UMR1093-CAPS, Université Bourgogne Franche-Comté, UFR des Sciences de Santé, Dijon, France; ^2^Service de Neurologie, CHRU Dijon, Dijon, France; ^3^EA4267 PEPITE, FHU INCREASE, University of Bourgogne Franche-Comté, Besançon, France

**Keywords:** BDNF, cognition, endothelium, NO, TrkB

## Abstract

Cognitive abilities are largely dependent on activation of cerebral tropomyosin-related kinase B receptors (TrkB) by brain-derived neurotrophic factor (BDNF) that is secreted under a bioactive form by both neurons and endothelial cells. In addition, there is mounting evidence for a link between endothelial function and cognition even though the underlying mechanisms are not well known. Therefore, we investigated the cerebral BDNF pathway, either neuronal or endothelial, in rheumatoid arthritis (RA) that combines both endothelial dysfunction (ED) and impaired cognition. Adjuvant-induced arthritis (AIA) in rats was used as a model of RA. Clinical inflammatory symptoms were evaluated from an arthritis score and brains were collected at day 31 ± 2 post-immunization. Neuronal expression of BDNF and TrkB phosphorylated at tyrosine 816 (p-TrkB) was examined in brain slices. Endothelial BDNF and p-TrkB expression was examined on both brain slices (hippocampal arterioles) and isolated cerebral microvessels-enriched fractions (vessels downstream to arterioles). The connection between endothelial nitric oxide (NO) and BDNF production was explored on the cerebrovascular fractions using endothelial NO synthase (eNOS) levels as a marker of NO production, *N*^ω^-Nitro-L-arginine methyl ester hydrochloride (L-NAME) as a NOS inhibitor and glyceryl-trinitrate as a slow releasing NO donor. Brain slices displayed lower BDNF and p-TrkB staining in both neurons and arteriolar endothelial cells in AIA than in control rats. For endothelial cells but not neurons, a strong correlation was observed between BDNF and p-TrkB staining. Of note, a strong correlation was also observed between neuronal p-TrkB and endothelial BDNF staining. In cerebral microvessels-enriched fractions, AIA led to decreased BDNF and eNOS levels with a positive association between the 2 parameters. These effects coincided with decreased BDNF and p-TrkB staining in endothelial cells. The exposure of AIA cerebrovascular fractions to GTN increased BDNF levels while the exposure of control fractions to L-NAME decreased BDNF levels. Changes in the cerebral BDNF pathway were not associated with arthritis score. The present study reveals that AIA impairs the endothelial and neuronal BDNF/TrkB pathway, irrespective of the severity of inflammatory symptoms but dependent on endothelial NO production. These results open new perspectives for the understanding of the link between ED and impaired cognition.

## Introduction

Brain-derived neurotrophic factor (BDNF) is present in high concentrations in the adult brain where it plays a crucial role in neuroplasticity, neurogenesis and angiogenesis (Pencea et al., 2001; Kim et al., 2004; Lu et al., 2014) through the phosphorylation of its cognate TrkB (tropomyosin-related kinase B) receptors at tyrosine 816 (p-TrkB) (Bathina and Das, 2015). Thus, decreased brain BDNF and TrkB levels in genetically-modified mice induced changes in behavioral deficit (Korte et al., 1995; Zorner et al., 2003), while transgenic mice overexpressing TrkB showed improvement in learning abilities (Koponen et al., 2004). It is generally assumed that low BDNF levels in the brain are due to a deficit in BDNF production by neurons as neurons are considered as the main source of BDNF in the brain. However, in opposition with this traditional thinking, we recently showed that the removal of endothelial cells from the brain resulted in a marked decrease in BDNF levels measured in the cerebral tissue (Monnier et al., 2017b). This shift of paradigm in the cellular origin of cerebral BDNF indicates that a hitherto unexpected large part of BDNF found in the brain corresponds to BDNF produced by cerebral endothelial cells and paves the way for the exciting hypothesis that neuronal function might be dependent not only on neuronal-derived BDNF but also on BDNF secreted by endothelial cells of cerebral capillaries. These data emphasize the importance to separately explore the brain BDNF/TrkB pathway at both the neuronal and endothelial level, especially since the literature continues to report a link between cardiovascular risk factors and impaired cognition (Knopman et al., 2001; Diener, 2011; Srinivasa et al., 2016).

Rheumatoid arthritis (RA) is a chronic autoimmune inflammatory joint disease associated with high cardiovascular risk (del Rincon et al., 2001) and impaired cognition including alteration in logical memory, memory working and executive function and presence of depressive symptoms (Bartolini et al., 2002; Sturgeon et al., 2016; Baptista et al., 2017). RA-associated increased cardiovascular risk has been related to endothelial dysfunction (ED) (Bergholm et al., 2002) and subsequent acceleration of atherosclerosis (Del Rincon et al., 2007). ED is present early in the course of the disease as evidenced from decreased brachial flow-mediated dilatation (Xu et al., 2017), which is a consequence of decreased endothelial NO production/activity in response to hyperemia. Studies in AIA rats identified mechanisms involved in decreased endothelial NO production/bioactivity including eNOS uncoupling (Haruna et al., 2006), arginase upregulation (Prati et al., 2011, 2012) and increased NO inactivation by oxidative stress (Haruna et al., 2006, 2007). On the contrary, the mechanisms involved in impaired cognition remain poorly understood even though a role of ED is suspected from studies reporting impaired cognition in patients exposed to cardiovascular risk factors. Surprisingly, while activation of cerebral TrkB by BDNF is largely involved in cognition, whether RA is associated with an impairment of cerebral BDNF pathway is unknown.

The present study investigated the cerebral BDNF pathway in rats subjected to adjuvant-induced arthritis (AIA) and the mechanisms involved in potential changes. The brains were collected at day 31 ± 2 post-immunization, time at which inflammatory symptoms are maximal and ED occurs at the peripheral macro- and micro-vasculature (Totoson et al., 2015). Neuronal BDNF and p-TrkB expression were examined on hippocampal brain slices. Endothelial BDNF and p-TrkB expression were examined on cerebral arterioles and downstream vessels using immunohistochemical analysis on hippocampal brain slices and Western blot analysis of isolated cerebral microvascular-enriched fractions, respectively. The potential connection between the cerebral BDNF pathway with the severity of inflammatory symptoms or endothelial NO production was also investigated.

## Methods

### Animals

Experiments were carried out on 6 weeks-old male Lewis rats (*n* = 64) that were purchased from Janvier (Le Genest Saint Isle, France). Experiments were conducted according to the French department of agriculture guidelines (license 21 CAE-102) and approved by the local ethic committee. The experimental procedures were performed in order to comply with ARRIVE guidelines. Animals were housed under a 12 h/12 h light/dark cycle and allowed free access to food and water. Anesthesia was induced by isoflurane 4% (Virbac, Carros, France) for arthritis induction and chloral hydrate anesthesia (400 mg/kg, i.p.; Sigma-Aldrich, Saint-Quentin-Fallavier, France) for brain removal.

### Induction and clinical evaluation of arthritis

Arthritis was induced by a single intradermal injection at the base of the tail of 120 μL of 1 mg of heat-killed *Mycobacterium butyricum* (Difco, Detroit, MI) suspended in 0.1 ml of mineral oil [Freund's incomplete adjuvant (Difco, Detroit, MI)]. Non-arthritis Lewis age-matched rats that were used as controls received 120 μL of saline. Indeed, Freund's incomplete adjuvant was previously suspected to interfere with the th1/th2 balance of immune response (Zhang et al., 1999). The clinical scoring system (arthritis score) of inflammation was employed as follows (Sakaguchi et al., 2003): inflammation (erythema and swelling) of one finger scores 0.1, weak and moderate arthritis of one big joint (ankle or wrist) scores 0.5 and intense arthritis of one big joint scores 1. Tarsus and ankle were considered as the same joint. Arthritis score for a given limb ranged from 0 to 1.5 and global arthritis score (4 limbs) ranged from 0 to 6. The arthritis score was regularly determined until animal sacrifice.

### Preparation of cerebral microvessels-enriched fractions

The procedure was detailed elsewhere (Monnier et al., 2017a). Briefly, after the removal of large superficial vessels, the forebrain except the hypothalamus was homogenized in ice cold Hank's balanced salt solution (HBSS) with a Potter-Thomas homogenizer. After centrifugation the pellet (P1) was saved and the supernatant was centrifuged again. The new pellet (P2) was pooled with P1, suspended in 20% dextran and centrifuged. The new pellet (P3) was again saved and the remaining tissue was reprocessed similarly, thus leading to P4. P3 and P4 were pooled together and suspended in HBSS. They were successfully filtered through a 335, 110, 53, and 20 μm mesh nylon filters. The fraction retained on the 335- and 110 μm-filters were discarded while fractions retained on other filters (F53 and F20) were kept for further analysis. We previously showed that cells from F53 and F20 were all positive for both BDNF and the endothelial marker GLUT1 (Monnier et al., 2017b), indicating that BDNF is constitutively expressed by endothelium of cerebral microvasculature.

### Western blot analysis

Pooled microvessels-enriched fractions (F53 + F20) were homogenized in ice-cold lysis buffer [100 mmol/L Tris-HCl (pH 7.4), 150 mmol/L NaCl, 1 mmol/L EGTA, 1% triton X-100, 1% protease inhibitor cocktail (P8340, Sigma-Aldrich, Saint-Quentin-Fallavier, France)]. After centrifugation of homogenates, an aliquot of the supernatant was kept for protein measurement by using the Lowry method. Equal protein amounts were resolved by SDS-PAGE and electrophoretically transferred to polyvinylidene difluoride (PVDF) membranes (0.2 μm) for western blotting. After blocking non-specific binding sites with a 5% solution of non-fat dry milk in TBS (20 mM Tris/HCl, 137 mM NaCl, pH 7.4) containing 0.1% Tween 20, membranes were probed with an anti-BDNF rabbit monoclonal antibody (1/3,000 with 5% non-fat dry milk, ab108319, Abcam, Cambridge, United Kingdom), an anti-eNOS mouse monoclonal antibody (1/2,500 with 5% non-fat dry milk, 610297, BD Biosciences, San Jose, USA) or an anti-β-actin antibody (1/10,000, A5441, Sigma-Aldrich, Saint Quentin-Fallavier, France). Then, membranes were incubated with secondary antibodies conjugated with horseradish peroxidase [111-035-144 (anti-rabbit, 1/20,000) and 115-035-166 (anti-mouse, 1/50,000), Jackson ImmunoResearch Laboratories, Interchim, Montluçon, France]. Protein-antibody complexes were visualized using the enhanced chemiluminescence Western blotting detection system (ECL 2, 1151-7371, Fisher Scientific, Illkirch, France). The band densities were determined by scanning densitometry (GS-800, BIO-RAD Laboratories, Ivry sur Seine, France). Whole membranes for BDNF and eNOS are available on supplemental data (Datasheet 1).

### Immunohistochemical analysis

Immunohistochemical experiments were performed on pooled microvessels-enriched fractions (F53 + F20) and brain slices. Vascular fractions were cryoprotected in HEPES/sucrose buffer, fixed in methanol and collected on superFrost Plus slides. For preparation of brain slices, brain of anesthetized rats was successively transcardially perfused with saline and 4% paraformaldehyde (PFA) solution in 0.1 M phosphate buffer (PB) (pH 7.4). The brains were then removed, post-fixed in PFA (1 h), cryoprotected with a sucrose solution (20% in PB for 48 h) and then transversally cryosectioned (20 μm-thick) with a cryostat (HM550, OMPV, Microm-Microtech, Francheville, France) at −20°C and collected on SuperFrost Plus slides. After fixation and blockade of non-specific binding sites, slices were first incubated with the rabbit monoclonal anti-BDNF antibody (dilution 1/200, ab108319, Abcam, Cambridge, United Kingdom) or anti-phospho-TrkB Y816 polyclonal antibody (dilution 1/200, ABN1381, Merck-Millipore, Saint Quentin en Yvelines, France), in the presence of antibodies directed against the specific endothelial markers, either GLUT1 (dilution 1/300, mouse monoclonal, MABS132, Merck-Millipore, Saint Quentin en Yvelines, France) or vWF(dilution 1/200, mouse monoclonal, MCA 3442, Merck-Millipore, Saint Quentin en Yvelines, France), or the specific neuronal marker NeuN (dilution 1/100, mouse monoclonal, MAB377, Merck-Millipore, Saint Quentin en Yvelines, France). They were then exposed to a fluorescent secondary antibody ALEXA Fluor 488 and 568 [A11029 (anti-mouse) and A11036 (anti-rabbit), Molecular Probes, Invitrogen, Cergy Pontoise, France, dilution 1/1,000]. Negative controls were prepared by omitting the primary antibodies. Finally, slides were mounted with DAPI (a nuclear marker)-containing mounting medium (Vectashield, FP-DT094A, Interchim, Montluçon, France) and analyzed by using an epifluorescent microscope (Eclipse E600, Nikon France S.A.S., Champigny-sur-Marne, France). Endothelial and neuronal staining for BDNF and p-TrkB Y816 was examined using an automated method (software Matlab, Mathworks, Natick, USA) as described in Kim et al. (2015). The Matlab program can be downloaded in: http://faculty.jsd.claremont.edu/jarmstrong/fquant/index.html. The calculated fluorescence intensity corresponded to the mean of intensity exhibited by adjacent cells that were selected by the experimenter by plotting a line through adjacent endothelial or neuronal cells. Endothelial staining was quantified on cerebral microvessels-enriched fractions vascular fractions as well as on brain slices. It was quantified on the smallest vessels (capillaries) for cerebrovascular fractions and on three arterioles (seen in transversal section) present between the *Cornu Ammonis 1* (CA1) and *dentate gyrus* for brain slices. Neuronal staining was quantified on brain slices from a line passing throughout neurons of the CA1 subfield.

### *Ex vivo* experiments

Fresh pooled microvessels-enriched fractions were immediately distributed into wells previously loaded with a culture medium (M199 medium supplemented with 0.1 mg/L L-glutamine, 10% fetal bovine serum, 2% glucose, 2% amino acids and 1% antibiotic solution). Microvessels-enriched fractions collected in AIA rats were incubated (24 h, in a 95% O_2_-5%CO_2_ atmosphere) with either 10 μM glyceryl trinitrate (GTN, MR107753, Merck-Millipore, Saint Quentin en Yvelines, France) as a slow-releasing NO donor that well mimics physiological endothelial NO production or saline. Fractions collected in control rats were in parallel incubated with either 5 mM of the eNOS inhibitor N^w^-nitro-L-arginine methyl ester hydrochloride (L-NAME on BDNF levels, N5751, Sigma-Aldrich, Saint-Quentin-Fallavier, France) or saline. At the end of the incubation period, cerebrovascular fractions were prepared for Western blotting analysis.

### Statistical analysis

Results were expressed as means ± standard deviations (SD). Statistical analysis between two groups was performed using a parametric *t*-test or non-parametric Mann–Whitney's test, depending on normality and equal variance tests. The relationship between two variables was investigated using Pearson's and Spearman's correlations (for normally or not normally distributed data, respectively). Differences were considered significant at *p* < 0.05.

## Results

### The neuronal BDNF/TrkB pathway is impaired in AIA

**Figure 1:**
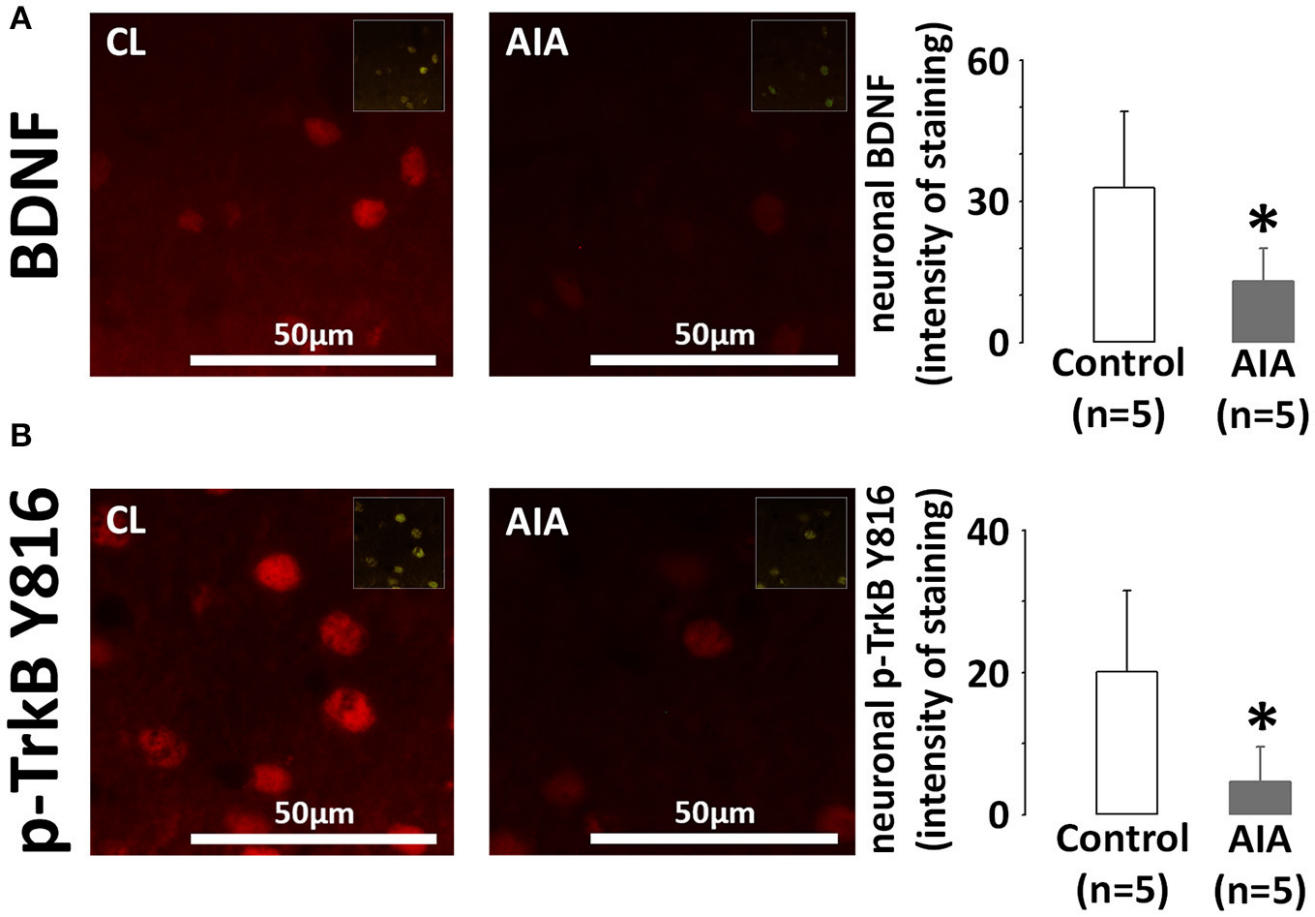
**(A)** Effect of AIA on neuronal BDNF expression using immunohistochemical analysis of brain slices. Representative photographs of BDNF (red) labeling in neurons of the CA1 hippocampal region and quantification of staining intensity, **(B)** Effect of AIA on neuronal p-TrkB Y816 receptors expression (see legend A). NeuN was used as a neuronal marker (merged images in inserts). Brain samples were collected at day 31 ± 2 post-immunization. Values are expressed as means ± SD, *n* = number of rats, ^*^p < 0.05 vs. control rats.

Brains were collected in AIA (*n* = 5, arthritis score = 3.6 ± 0.8) and control rats (*n* = 5). BDNF and p-TrkB expression was examined on hippocampal slices at the level of the CA1 region. Indeed, the hippocampus is a cognition-related brain region. In addition, the low neuronal densitiy of the CA1 subfield offers the opportunity to easily quantify the neuronal fluorescence intensity. As shown in Figure 1A, AIA decreased BDNF staining in cells positive for the neuronal marker NeuN, the intensity of staining in control and AIA rats being 32.8 ± 16.3 and 13.0 ± 7.0, respectively. In these neurons, p-TrkB staining (Figure 1B) was also lower in AIA rats than in controls, the intensity of staining dropping from 20.1 ± 11.4 in control rats to 4.6 ± 4.9 in AIA rats. However, no association was observed between neuronal BDNF and p-TrkB. staining (*r*_s_ = 0.428, *p* = 0.21, data not shown) when control and AIA were simultaneously observed.

### The endothelial BDNF/TrkB pathway is impaired in AIA

**Figure 2:**
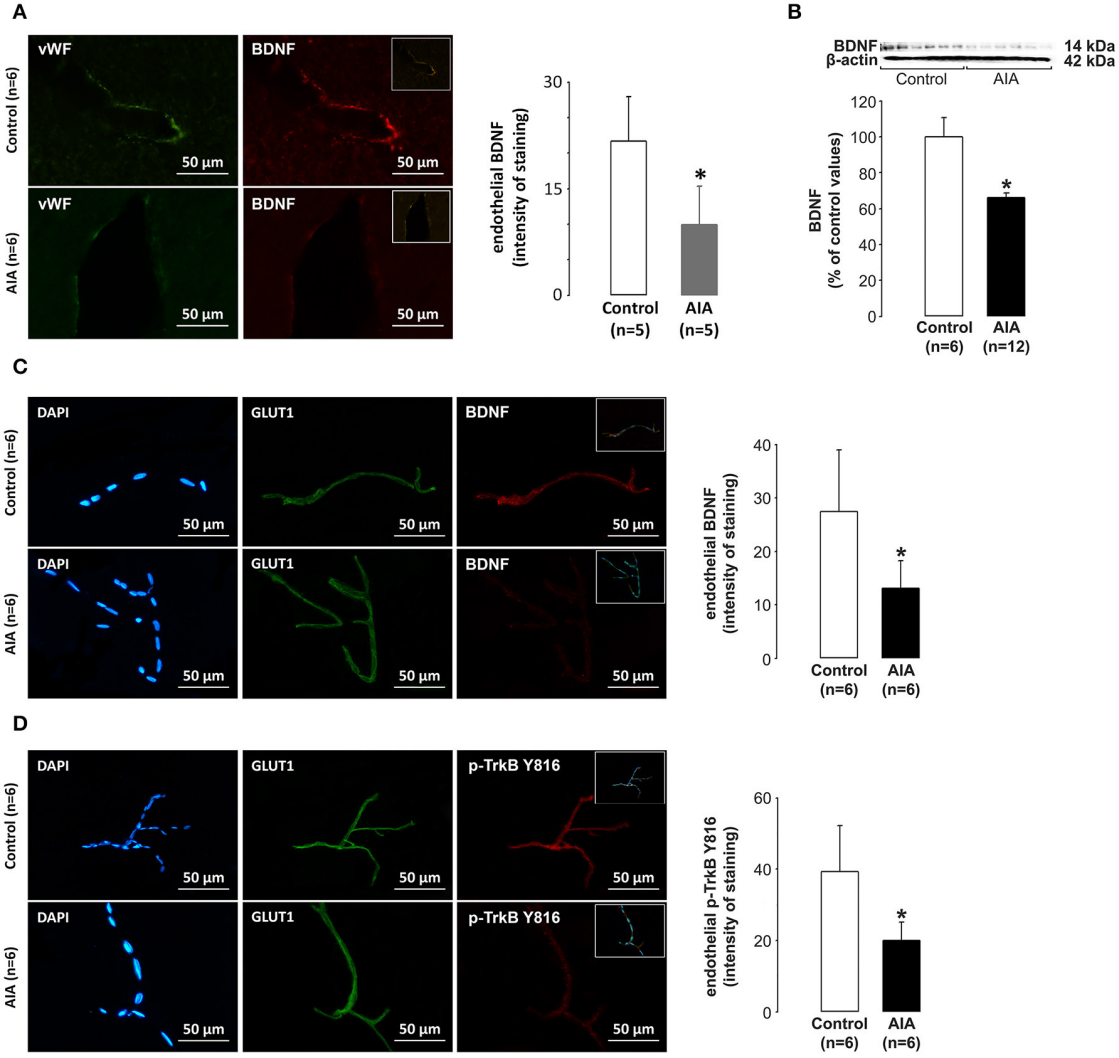
**(A)** Effect of AIA on endothelial BDNF expression using immunohistochemical analysis of brain slices. Representative photograph of vWF (a marker of endothelial cells, green), BDNF (red) labeling and merged images (insert) in hippocampal arterioles and quantification of endothelial staining intensity, **(B)** Effect of AIA on BDNF levels in cerebral microvessels-enriched fractions. A representative immunoblot of BDNF and β-actin as internal control is shown above the bar graphs, **(C)** Effect of AIA on endothelial BDNF expression using immunohistochemical analysis of cerebral microvessels-enriched fractions. Representative photographs of DAPI (a nuclear marker, blue), GLUT1 (a marker of endothelial cells, green) BDNF (red) immunostaining and merged images (inserts) and quantification of staining intensity, **(D)** Effect of AIA on endothelial p-TrkB Y816 receptors expression (see legend **C**). Brain and vascular samples were collected at day 31 ± 2 post-immunization. Values are expressed as means ± SD, *n* = number of rats, ^*^p < 0.05 vs. control rats.

Arteriolar endothelial BDNF expression was first examined on brain slices used for investigation of the neuronal BDNF pathway. We focused on arterioles (seen in transversal section) located between the *Cornu Ammonis 1* (CA1) and *dentate gyrus* of the hippocampus. As shown in Figure 2A, BDNF labeling in cells lining the arteriolar lumen (cells positive for the endothelial marker vWF) was lower in AIA (*n* = 5) than in control rats (*n* = 5). Then, we investigated the endothelial BDNF pathway in vessels dowstream to arterioles. For this purpose, the endothelial BDNF pathway was examined on cerebral microvascular-enriched fractions isolated from additional AIA (*n* = 18, 12 rats for Western blot analysis and 6 rats for immunohistochemical analysis) and control rats (*n* = 12). According to Western blot analysis, 6 control and 6 AIA rats (arthritis score = 2.8 ± 1.0) were run on a same membrane, the remaining AIA rats (arthritis score = 1.5 ± 0.9) being run on another membrane with the same 6 control rats. BDNF values in AIA rats were expressed as percentage of control values. As shown in Figure 2B, BDNF levels in cerebral microvascular-enriched fractions (F20 + F53) were significantly lower in AIA than in control rats (*n* = 6). These changes coincided with lower BDNF and p-TrkB staining in endothelial cells (cells positive for the specific endothelial marker GLUT1) in AIA (*n* = 6, arthritis score = 3.3 ± 1.0) than in control rats (*n* = 6). Indeed, the intensity of endothelial BDNF staining at capillary levels was 27.4 ± 11.6 in control and 13.1 ± 5.2 in AIA rats (Figure 2C), the corresponding values for p-TrkB staining being 39.3 ± 13.0 and 20.1 ± 5.0 (Figure 2D). Notably, DAPI (a specific nuclear) labeling of (F20 + F53) fractions showed that these fractions were well enriched in capillaries (vascular wall consisting with a monolayer of endothelial cells) (see Figures 2C,D).

**Figure 3:**
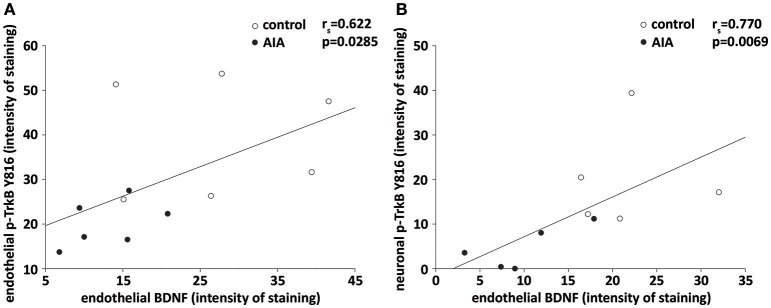
**(A)** In cerebral microvessels-enriched fractions, endothelial BDNF and p-TrkB Y816 staining were correlated when AIA (*n* = 6) and control rats (*n* = 6) were simultaneously examined, **(B)** on brain slices, endothelial BDNF staining of hippocampal arterioles correlated with p-TrkB Y816 staining in CA1 neurons when AIA (*n* = 5) and control rats (5) were simultaneously examined. *r*_s_ = spearman correlation coefficient.

Finally, we investigated to what extent endothelial BDNF could act as an autocrine and/or paracrine actions. Consistent with an autocrine action, a positive correlation was observed between endothelial BDNF and p-TrkB staining in cerebral microvessels-enriched when control and AIA were simultaneously analyzed (Figure 3A). A positive correlation was also observed between endothelial BDNF staining of hippocampal arterioles and p-TrkB staining by CA1 neurons when the two groups of rats were again simultaneously analyzed, suggesting a paracrine action of endothelial BDNF on neurons (Figure 3B).

### No controls BDNF production by endothelial cells in AIA

**Figure 4:**
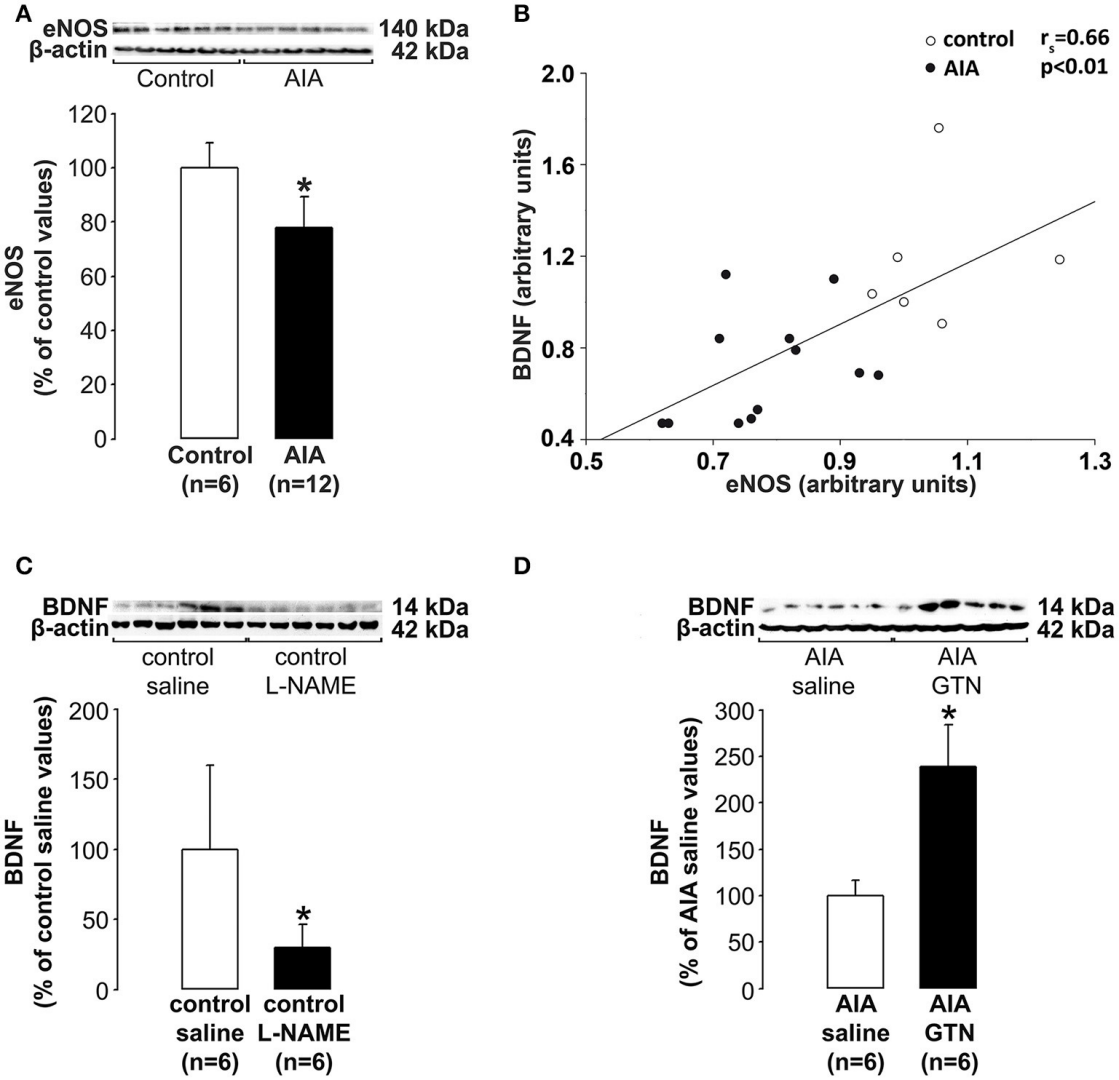
**(A)** Effect of AIA on eNOS levels in cerebral microvessels-enriched fractions. A representative immunoblot of eNOS and β-actin as internal control is shown above the bar graphs, **(B)** In these fractions, eNOS and BDNF levels correlated, **(C)** Effect of the exposure of cerebral microvessels-enriched fractions collected in control rats to N^w^-nitro-L-arginine methyl ester hydrochloride (L-NAME) on BDNF levels, **(D)** Effect of the exposure of cerebral microvessels-enriched fractions collected in AIA rats to glyceryl-trinitrate (GTN) on BDNF levels. Vascular samples were collected at day 31 ± 2 post-immunization. Values are expressed as means ± SD, *n* = number of rats, ^*^p < 0.05 vs. corresponding controls.

While it is well documented that AIA led to decreased endothelial NO bioactivity in peripheral vessels (see introduction) whether the production of NO by the cerebral endothelium is altered by AIA has never been investigated. Therefore, we measured eNOS levels in cerebrovascular fractions (previously used to investigate BDNF levels) as an indicator of NO production by the cerebral endothelium. As shown in Figure 4A, eNOS levels were significantly lower in AIA than in control rats. Then, we explored the connection between levels of eNOS and BDNF in cerebral microvessels-enriched fractions. As shown in Figure 4B, a positive correlation was found between the 2 parameters when control and AIA rats were examined simultaneously. By contrast, no association was found when AIA and control rats were examined separately (data not shown). Finally, cerebrovascular fractions were isolated from 24 additional rats (12 controls and 12 AIA). The fractions we exposed (24 h) isolated vascular fractions to L-NAME (a NOS inhibitor) or GTN (a slow releasing NO donor that well mimics the continuous secretion of NO by endothelial cells). The exposure to L-NAME (μM) of control fractions was found to induce a significant decrease in BDNF levels (Figure 4C), while the exposure to GTN (10 μM) of AIA fractions increased BDNF levels (Figure 4D). Collectively, these data suggest that decreased BDNF production by the cerebral endothelium was likely consecutive to its reduced capacity to produce NO.

### Changes in either the cerebral BDNF pathway or eNOS levels evoked by AIA did not proportionate with the severity of inflammatory symptoms

**Figure 5:**
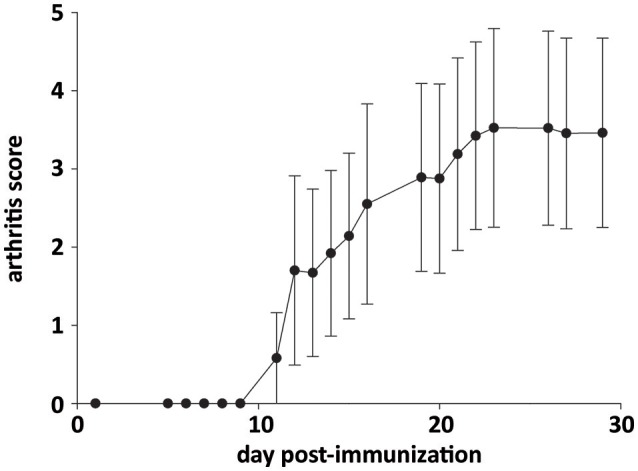
Time course of arthritis score in AIA rats. Values are expressed as means ± SD of 35 rats.

The severity of inflammatory symptoms was evaluated from arthritis scores in all AIA rats (*n* = 35). As shown in Figure 5, the first symptoms were seen ~ at day 12 post-immunization. Then, arthritis score rapidly increased to 3.5 and plateaued from ~ day 22 post-immunization until sacrifice. Notably, arthritis score at sacrifice differed among rats, its minimum and maximum value being 0.7 and 4.9, respectively. However, when individual arthritis score in AIA rats was plotted against corresponding values of BDNF or eNOS levels in cerebral microvessels-enriched fractions or intensity of neuronal BDNF staining on brain slices, no correlation was observed (Table 1).

**Table 1:** AIA-induced changes in the BDNF pathway and endothelial NO production do not relate to the severity of inflammatory symptoms.

	**Arthritis score**	
	***r***	***P***
Vascular BDNF levels/athritis score serie 1	−0.005	0.993
Vascular BDNF levels/arthristis serie 2	−0.653	0.160
Vascular eNOS levels/arthritis score serie 1	0.445	0.376
Vascular eNOS levels/arthritis score serie 2	0.123	0.816
Neuronal BDNF staning/arthritis score	−0.416	0.486

*Arthritis score as reflection of the severity of inflammatory symptoms was determined in AIA rats just before brain collection (at day 31 ± 2 post-immunization). Arthritis score was plotted against corresponding BDNF or eNOS levels (Western blot analysis, n = 6 for series 1 and 2) in cerebral microvessels-enriched fraction or corresponding neuronal BDNF staining intensity (immunohistochemical analysis of brain slices, n = 5). r = Pearson's correlation coefficient. n = number of AIA rats. No correlation was observed between arthritis score and other parameters*.

## Discussion

The main results of the present study are that (i) AIA alters the cerebral BDNF/TrkB pathway at both the endothelial and neuronal levels, (ii) activation of neuronal TrkB receptors correlated with endothelial BDNF expression, (iii) a connection exists between the endothelial BDNF/TrkB pathway and the capacity of the cerebral endothelium to produce NO, (iv) AIA-induced changes in either the cerebral BDNF pathway or endothelial NO production did not proportionate with the severity of inflammatory symptoms.

The impairment of the cerebral BDNF pathway is largely involved in impaired cognition associated in animal models of psychiatric, neurologic and neurodegenerative diseases. However, whether changes preferentially occur at the neuronal and/or endothelial level is not well known. In fact, levels of BDNF and its receptors are commonly measured in brain homogenates and their localization restricted to neurons. The new data provided by the present study is that AIA alters the cerebral BDNF/TrkB pathway at both the neuronal and endothelial level. More precisely, neuronal as well as endothelial expression of BDNF and activated TrkB receptors were lower in the brain of AIA rats than in that of controls, suggesting that impaired BDNF-dependent cognition might have both a neuronal and endothelial component. The assessment of cognitive deficit (memory, learning) by traditional behavioral tests in AIA rats is challenged by the reduction of spontaneous mobility as a result of inflammation of the two hind paws. However, learning is impaired in animal model of RA in which only one hind paw is damaged (Zhu et al., 2016). It is noteworthy that diseases other than RA but combining as RA impaired cognition and high cardiovascular risk such as hypertension and diabetes were reported to induce BDNF downregulation in neurons (Pietranera et al., 2010; Franco-Robles et al., 2014), endothelial cells (Navaratna et al., 2011; Prigent-Tessier et al., 2013) or both (Monnier et al., 2017a). Using cell culture, activation of endothelial TrkB receptors was reported to induce myotubes formation (angiogenesis) (Kim et al., 2004), while activation of neuronal receptors was reported to increase the number of synaptic connections (Park and Poo, 2013). Furthermore, BDNF secreted by cerebral endothelial cells in culture was reported to induce neurogenesis (Leventhal et al., 1999) and protect neurons against cytotoxic stimuli (Guo et al., 2008, 2012). The emerging and elusive question concerns the contribution of BDNF secreted by cerebral capillaries to neuronal function *in vivo* and by extension to cognition. Supportive such a paracrine action of endothelial-derived BDNF *in vivo*, a strong positive correlation was observed between endothelial BDNF and neuronal p-TrkB expression even though BDNF expression by capillaries was estimated from BDNF expression by arteriolar endothelial cells. We are aware that endothelial BDNF expression in arterioles and cerebral capillaries might differ. However, the difference if present is expected to be minor since endothelial BDNF staining/levels did not singificantly differ amoung F20, F53, and F110 fractions (Monnier et al., 2017a). According to such a paracrine action, BDNF secreted by cerebral capillaries into the cerebral interstitial fluid might be recognized by and activate neuronal TrkB receptors thereby increasing synapses strength and cognitive abilities. Although still speculative, such a scenario fits well with the new concept that emphasizes the importance of endothelial health in maintaining neuronal function. Last but not the least, our results showed that AIA-induced changes in the cerebral BDNF pathway, either neuronal or endothelial, were not in proportion with the severity of inflammatory symptoms. Further studies are however needed to investigate the impact of effective anti-rheumatoid treatment on the cerebral BDNF pathway.

Decreased endothelial NO bioactivity is well documented in RA patients (Steyers and Miller, 2014) and AIA rats (Haruna et al., 2006, 2007; Prati et al., 2011, 2012; Totoson et al., 2015), at least for the peripheral vasculature. Such alteration in endothelial phenotype and subsequent stimulation of atherosclerosis has been largely involved in the 2–5 times increased risk of developing premature cardiovascular diseases in RA (Wallberg-Jonsson et al., 1997). The new data provided by the present study is that ED extends to the cerebral microvasculature in AIA as evidenced by lower eNOS levels in cerebral enriched-microvascular fractions in AIA than in control rats. A clinically-relevant data is that vascular eNOS levels in AIA were not in proportion with the severity of inflammatory symptoms, suggesting that cerebral ED in RA patients cannot be predicted from the disease severity as previously observed for peripheral ED (Bernelot Moens et al., 2016). We are aware that decreased eNOS levels is only one of the multiple mechanisms that can lead to decreased endothelial NO bioactivity in AIA. Unfortunately, additional mechanisms cannot be explored in the present study because the amount of vascular material that can be purified from each rat forebrain was too low (250 μg total protein). We prefer to give the priority to the simultaneous measurement of eNOS and BDNF protein levels in the same sample, thus offering the opportunity to explore the link between endothelial NO and BDNF production. It is noteworthy that a positive correlation was observed between vascular eNOS and BDNF levels. These data combined with our *ex vivo* experiments showing that eNOS inhibition reproduced while NO supplementation reversed the effect of AIA on endothelial BDNF levels strongly suggest that decreased endothelial BDNF synthesis observed in AIA might be due to decreased capacity of the cerebral endothelium to synthesize NO. Notably, a positive control by endothelium-derived NO of BDNF synthesis by cerebral endothelial cells is not surprising. Indeed, the exposure of cultured brain-derived endothelial cells to inhibitors of NO production including the peptide A beta (a compound largely involved in the physiopathology of Alzheimer disease) and ADMA (an endogenous eNOS inhibitor) was reported to decrease BDNF production (Guo et al., 2008; Ma et al., 2015). In the same vein, incubation of isolated cerebral microvessels with advanced glycation-end products (Navaratna et al., 2011) that act as NO scavengers (Bucala et al., 1991) resulted in BDNF downregulation whilst their incubation with a slow releasing NO donor was recently reported by our laboratory to increase BDNF production (Monnier et al., 2017b). The new data provided by the present study is that a NO supplementation is efficient to stimulate endothelial BDNF production even when ED is present. A therapeutic perspective is that impaired cognition in RA might be improved by the restoration of endothelial NO production. However, even though our *ex vivo* experiments support a causal link between decreased endothelial NO production and BDNF production in AIA, a contribution of other factors cannot be excluded. In addition, whether neuronal BDNF expression, which is mainly controlled by neuronal activity (Balkowiec and Katz, 2000), is dependent on endothelial-derived BDNF remains an open question (Banoujaafar et al., 2016). An unresolved point concerns the role of pro-inflammatory in AIA-induced changes in BDNF. However, against their involvement the exposure to tumor necrosis factor alpha (TNFα), a cytokine relevant in RA, was previously reported to induce BDNF upregulation either by cultured cerebral endothelial cells (Bayas et al., 2002) or neuron-enriched dissociated culture of trigeminal ganglion (Balkowiec-Iskra et al., 2011). Nevertheless, assuming that cognition may be dependent on endothelial-derived BDNF (Katusic and Austin, 2014), a therapeutic perspective of the present study is that impaired cognition in RA might be improved by the restoration of endothelial NO production. Consistent with this new approach, prevention of ED was recently reported to translate into brain BDNF levels elevation in AIA rats (Pedard et al., 2017).

In conclusion, the present study supports the exciting hypothesis that decreased BDNF production by the cerebral endothelium as a result of decreased endothelial NO synthesis might account for AIA-associated decreased activation of neuronal TrkB activation. These data open new perspectives in the comprehension of the link between endothelial function and cognition and in the management of impaired cognition in patients at high risk for cardiovascular diseases.

## Author contributions

All authors were involved in drafting the article, and all authors approved the final version to be published. CM had full access to all of the data in the study and takes responsibility for the integrity of the data and the accuracy of the data analysis. Study conception and design: CD, CM. Acquisition of data: MP, AQ, AT, PG. Analysis and interpretation of data: MP, CD, CM.

### Conflict of interest statement

The authors declare that the research was conducted in the absence of any commercial or financial relationships that could be construed as a potential conflict of interest.
