# Supplementary material for: The Cerebral Brain-Derived Neurotrophic Factor Pathway, Either Neuronal or Endothelial, Is Impaired in Rats with Adjuvant-Induced Arthritis. Connection with Endothelial Dysfunction
Source: Front Physiol. 2018 Jan 9;8:1125. doi: 10.3389/fphys.2017.01125 (PMC5767301; doi:10.3389/fphys.2017.01125)
Supplement: Supplementary file 1 [file DataSheet1.DOCX]

**Supplemental data**


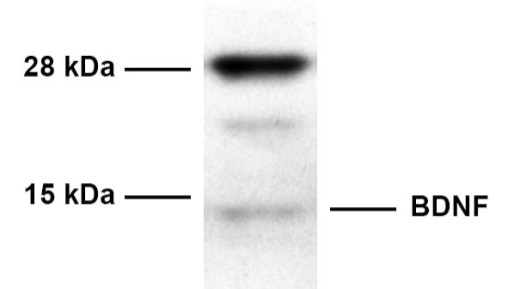


**Figure 1 Supplemental data.** Representative BDNF immunoblot from microvessels-enriched fractions (prepared from 1 control rat). Wells were loaded with 20 µg of protein. BDNF (the mature form) corresponded to the band at 14 kDa.


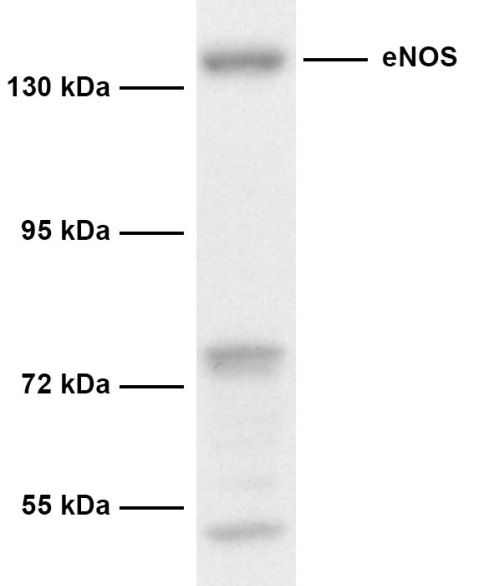


**Figure 2 Supplemental data.** Representative eNOS immunoblot from microvessels-enriched fractions (prepared from 1 control rat). Wells were loaded with 35 µg of protein. eNOS corresponded to the band at 140 kDa.
